# *Geosmithia*-*Ophiostoma*: a New Fungus-Fungus Association

**DOI:** 10.1007/s00248-017-1062-3

**Published:** 2017-09-05

**Authors:** Alessia L. Pepori, Priscilla P. Bettini, Cecilia Comparini, Sabrina Sarrocco, Anna Bonini, Arcangela Frascella, Luisa Ghelardini, Aniello Scala, Giovanni Vannacci, Alberto Santini

**Affiliations:** 1 Institute for Sustainable Plant Protection (IPSP-CNR), via Madonna del Piano 10, 50019 Sesto Fiorentino, FI Italy; 2 Department of Biology, University of Florence, via Madonna del Piano 6, 50019 Sesto Fiorentino, FI Italy; 3 Department of Agri-Food Production and Environmental Science (DiSPAA), University of Florence, Piazzale delle Cascine 28, 50144 Florence, Italy; 4 Department of Agriculture, Food and Environment (DAFE), University of Pisa, via del Borghetto 80, 56124 Pisa, Italy

**Keywords:** Biological control, Dutch elm disease (DED), Fungus-fungus interaction, *Geosmithia* spp., Mycoparasite, *Ophiostoma novo-ulmi*

## Abstract

In Europe as in North America, elms are devastated by Dutch elm disease (DED), caused by the alien ascomycete *Ophiostoma novo-ulmi*. Pathogen dispersal and transmission are ensured by local species of bark beetles, which established a novel association with the fungus. Elm bark beetles also transport the *Geosmithia* fungi genus that is found in scolytids’ galleries colonized by *O. novo-ulmi*. Widespread horizontal gene transfer between *O. novo-ulmi* and *Geosmithia* was recently observed. In order to define the relation between these two fungi in the DED pathosystem, *O. novo-ulmi* and *Geosmithia* species from elm, including a GFP-tagged strain, were grown in dual culture and mycelial interactions were observed by light and fluorescence microscopy. Growth and sporulation of *O. novo-ulmi* in the absence or presence of *Geosmithia* were compared. The impact of *Geosmithia* on DED severity was tested in vivo by co-inoculating *Geosmithia* and *O. novo-ulmi* in elms. A close and stable relation was observed between the two fungi, which may be classified as mycoparasitism by *Geosmithia* on *O. novo-ulmi*. These results prove the existence of a new component in the complex of organisms involved in DED, which might be capable of reducing the disease impact.

**Electronic supplementary material:**

The online version of this article (10.1007/s00248-017-1062-3) contains supplementary material, which is available to authorized users.

## Introduction

*Every species is intricately involved with a myriad of associates—some obligate, some facultative—that profoundly influence their evolution, physiology, and life history* [1]*.*

Dutch elm disease (DED) is a highly destructive vascular disease, which caused an extensive loss of mature elms in Europe, Asia, and North America during the twentieth century. The disease is caused by fungi of the genus *Ophiostoma* (*Ascomycota*, *Ophiostomatales*) and, in particular, by *Ophiostoma ulmi* (Buisman) Nannf and *Ophiostoma novo-ulmi* Brasier (ONU) [2]. Pathogen spreading and infection of suitable hosts are mainly ensured by elm bark beetles (EBB) (*Coleoptera*: *Curculionidae*, *Scolytinae*) [3]. The synchrony between the life cycles of host tree, fungus, and EBB allows vectors to disseminate ONU when host plants are most prone to infection and temperatures favorable for fungal growth, thus boosting the pathogen’s aggressiveness [4]. Moser et al. [5] showed that phoretic mites carried by EBB in turn transport ONU conidia, ascospores, and in some cases hyphae attached to their body surfaces, in sporothecae and in the digestive system. Mites may therefore contribute to DED transmission by spreading the fungus within the gallery system, enhancing ONU sexual reproduction and promoting an increase in genetic diversity through the fertilization of proto-perithecia. Moreover, mites may contribute to increase the spore load beyond the threshold required for infection [5].

The virulence of ONU might be negatively affected by the presence of a family of naturally occurring viruses, known as “d-factors,” found in the fungus cytoplasm [6] and able to prevent ONU from infecting healthy elms [7]. *O. novo-ulmi* isolates carrying these viruses exhibit slow, ragged growth, as well as a reduction in sporulation, perithecia production, and viability of conidia [8, 9]. In Europe, the virus was prevented from spreading into the ONU population, probably via the acquirement of the sexual compatibility type (MAT-1) and vegetative compatibility (*vic*) loci from *O. ulmi* [10]. Sexual reproduction alone helps to eliminate virus infection in ONU [11] leading to a rapid increase in the diversity of vegetative compatibility phenotypes [12], which reduces virus transmission.

The DED pathosystem is therefore a complex of interactions involving several other organisms in addition to the host plant, the pathogen, and the vector, ideally the entire EBBs’ holobiont [1] as well as the d-factor viruses, whose interplay influences the outcome of the infection.

EBB also transport species of the genus *Geosmithia* (*Ascomycota*: *Hypocreales*) [13], a monophyletic morphogenus of anamorphic ascomycetes that currently includes 32 published and at least 20 unpublished species of mitosporic fungi [14–19]. *Geosmithia* fungi may live as saprobes on various plant substrates, in soil or foodstuffs, and as true plant endophytes and are in most cases insect-associated [14, 17, 19, 20]. Several *Geosmithia* species have, at least for some parts of their lives, the same habitat as ONU on dying elms, although they occupy different ecological niches [21].

The existence of a more complicated relationship between ONU and Geosmithias than just occupying the same habitat and having the same vectors has recently been suggested by the discovery of widespread horizontal gene transfer (HGT) of a genomic fragment comprising the cerato-ulmin (*cu*) gene between the two fungi [22]. Cerato-ulmin, a class II hydrophobin of about 8 kDa produced by the pathogens *O. ulmi*, *O. novo-ulmi*, *Ophiostoma himal-ulmi*, and by the non-pathogen *Ophiostoma quercus* [23, 24], might play a role in DED by improving the fitness of the fungus [25].

Several hypotheses were made on the ecological role of *Geosmithia* spp. on host trees, but no conclusive evidence has been provided [17, 20, 26]. The aim of the present study was to examine the occurrence of a relationship between *Geosmithia* spp. and some Ophiostomatoid fungal species that have the same host plants and vectors and to define the nature of this relationship. The potential consequences of such a relationship on the DED pathosystem are also discussed.

The study focused on the “*elm system*,” comprising species of *Ophiostoma* and *Geosmithia* specific to elms. The *elm system* was put in comparison with systems comprising *Geosmithia* and Ophiostomatoid species from other host plants as oak and conifers (“*non-elm systems*”).

## Materials and Methods

### Fungal Strains and Media

The fungal species and strains included in this study are reported in Table 1. For the sake of brevity, in this paper, the term Ophiostomatoid fungi will be indistinctly used to refer to fungal species in orders *Ophiostomatales* and *Microascales* that share morphological analogies as the result of convergent evolution due to their association with insect vectors [28].

**Table 1:** *Geosmithia* and Ophiostomatoid fungi strains used in this work. In particular: experiment a, fungal growth rate in dual culture; experiment b, stereoscopic examination of mycelial interactions; experiment c, observation of hyphal interaction in white light microscopy; experiment d, transformation of *Geosmithia* strain with the GFP gene; experiment e, observation of hyphal interactions in fluorescence microscopy; experiment f, fertility test; experiment g, pathogenicity test

	Species	Strain no.	Source	Geographic origin	Year	Provided by	Reference	Experiment
*Ophiostoma novo-ulmi*	*O. novo-ulmi* ssp. *novo-ulmi* mtA	H327	*Ulmus* spp.	Brezno-Nizke, Tatry, Slovakia	1979	Brasier CM	Pipe et al. (1995)[30]	a, b, c, e, f
	*O. novo-ulmi* ssp. *novo-ulmi* mtB	H328	*Ulmus* spp.	Gottschalf Charkov, Russia	1979	Brasier CM	Brasier (1986)[8]	a, b, f, g
	*O. novo-ulmi* ssp. *americana* mtA	H172	*Ulmus* spp.	Keene, New Hampshire, USA	1977	Brasier CM	Pipe et al. (1995)[30]	a, b, c, e, f
	*O. novo-ulmi* ssp. *americana* mtB	H363	*Ulmus* spp.	Caleodon, Ireland	1980	Brasier CM	Brasier, (1986)[8]	a, b, f
*Ophiostoma ulmi*	*Ophiostoma ulmi* mtA	R21	*Ulmus* spp.	Bozovici, Romania	1986	Brasier CM	Pipe et al. (1995)[30]	b, c, e
	*Ophiostoma ulmi* mtB	E2	*Ulmus* spp.			Brasier CM	Gibbs et al. (1975)[31]	b, e
*Ophiostoma quercus*	*Ophiostoma quercus* mtA	CTK2-s	*Taphrorychus bicolor* on *Fagus sylvatica*	Lainzer Tiegarten, Austria	1995	Kirisits T	BOKU collection	a, b, f
	*Ophiostoma quercus* mtA	CTK117-s	*Taphrorychus bicolor* on *Fagus sylvatica*	Lainzer Tiegarten, Austria	1995	Kirisits T	BOKU collection	f
	*Ophiostoma quercus* mtB	CTK118-s	*Taphrorychus bicolor* on *Fagus sylvatica*	Lainzer Tiegarten, Austria	1995	Kirisits T	BOKU collection	f
	*Ophiostoma quercus* mtA	CTK120-s	*Platypus cylindrus* on *Fagus sylvatica*	Lainzer Tiegarten, Austria	1995	Kirisits T	BOKU collection	f
	*Ophiostoma quercus* mtA	CTK121-s	*Taphrorychus bicolor* on *Fagus sylvatica*	Lainzer Tiegarten, Austria	1995	Kirisits T	BOKU collection	a, b, f
	*Ophiostoma quercus* mtB	RZ/7-s	*Vitis vinifera*	Rhrendorf, Austria	2000	Kirisits T	BOKU collection	a, b
	*Ophiostoma quercus* mtA	TB/35-s	*Fagus sylvatica*	Lainzer Tiegarten, Austria	1995	Kirisits T	BOKU collection	a, b, f
Ophiostomatoid	*Ophiostoma* cf. *picea*	AT30-s	*Ulmus glabra*		1997	Kirisits T	BOKU collection	a, b
	*Ophiostoma kryptum*	Hasd/3	*Tetropium gabrieli* on *Larix decidua*	Austria	1995	Kirisits T	CBS 116182	a, b
	*Ophiostoma* cf. *clavatum*	AC/1/1/1	*Ips acuminatus* on *Pinus sylvestris*	Bleiberg, Austria		Kirisits T	BOKU collection	a, b
	*Ceratocystis polonica*	KOW/Ku/41	*Ips typographus* on *Picea abies*	Lower Austria, Rothwald, Austria	1997	Kirisits T	BOKU collection	a, b
	*Ceratocystis* cf. *minuta*	KW/3/4	*Picea abies*	Bialowieza, Poland	2002	Kirisits T	CBS 109966	a, b
	*Leptographium* sp.1	KW/2/2/2/1				Kirisits T	BOKU collection	a, b
	*Ophiostoma ainoae*	KW/Ku/29	*Picea abies*	Lower Austria, Kreisbach, Austria	1998	Kirisits T	BOKU collection	a, b
	*Ophiostoma tetropii*	CBS428.94	*Tetropium* sp. on *Picea abies*	Tyrol, Ehrwald, Austria	1994	Kirisits T	CBS 428.94	a, b
	*Graphium fimbrisporum*	R/4/1/2	*Picea abies*	Lower Austria, Hiesberg, Melk, Austria	1998	Kirisits T	CBS 421.94	a, b
	*Grosmannia piceiperda*	KW/4/2/2/1				Kirisits T	BOKU collection	a, b
	*Grosmannia penicillata*	KW/4/2/6/2	*Picea abies*	Salzburg, Austria	2003	Kirisits T	CBS 109990	a, b
*Geosmithia* from elms	*Geosmithia flava*	CNR120	*Ulmus minor*	Marsovice, Czech Rep.	2009	Pepori AL	Pepori et al. (2015)[21]	b, c, f
	*Geosmithia flava*	MK1551	*Pteleobius vittatus* on *Ulmus laevis*	Forest near Bulhary, Břeclav, Czech Rep.	2006	Kolařík M	Kolařík et al. (2008)[17]	f
	*Geosmithia langdonii*	MK1643	*Scolytus multistriatus* on *Ulmus laevis*	Cerninovsko, Neratovice, Czech Rep.	2005	Kolařík M	Kolařík et al. (2008)[17]	a, b, c, f
	*Geosmithia langdonii*	MK1644	*Scolytus multistriatus* on *Ulmus laevis*	Cerninovsko, Neratovice, Czech Rep.	2005	Kolařík M	Kolařík et al. (2008)[17]	f
	*Geosmithia langdonii*	MK1645	*Scolytus multistriatus* on *Ulmus laevis*	Cerninovsko, Neratovice, Czech Rep.	2005	Kolařík M	Kolařík et al. (2008)[17]	f
	*Geosmithia langdonii*	MK1646	*Scolytus multistriatus* on *Ulmus laevis*	Cerninovsko, Neratovice, Czech Rep.	2005	Kolařík M	Kolařík et al. (2008)[17]	a, f, g
	*Geosmithia omnicola*	CNR8	*Ulmus laevis*	Libick Luh Velky Osek, Czech Rep.	2009	Pepori AL	Pepori et al. (2015)[21]	b, c, f
	*Geosmithia omnicola*	MK544	*Pteleobius vittatus* on *Ulmus* spp.	Bakony Mts., Hungary	2003	Kolařík M	Kolařík et al. (2007)[16]	f
	*Geosmithia* sp. 2	CNR28	*Ulmus minor*	Strèdokluky, Czech Rep.	2009	Pepori AL	Pepori et al. (2015)[21]	b, c, f
	*Geosmithia* sp. 2	MK1638	*Scolytus multistriatus* on *Ulmus laevis*	Aracena, Andalusia, Spain	2005	Kolařík M	Kolařík et al. (2008)[17]	f
	*Geosmithia* sp. 2	MK1622	*Scolytus kirschii* on *Ulmus minor*	Jorairatar, Andalusia, Spain	2005	Kolařík M	Kolařík et al. (2007)[16]	f
	*Geosmithia* sp. 2	MK1623	*Scolytus kirschii* on *Ulmus minor*	Jorairatar, Andalusia, Spain	2005	Kolařík M	Kolařík et al. (2007)[16]	f
	*Geosmithia* sp. 20	CNR132	*Ulmus* FL364	Florence, Italy	2009	Pepori AL	Pepori et al. (2015)[21]	b, c, f
	*Geosmithia* sp. 23	MK896	Different *Ulmus* insects species on *Ulmus laevis*	Kančí obora forest, Břeclav, Czech Rep.	2005	Kolařík M	Kolařík et al. (2008)[17]	f
	*Geosmithia* sp. 5	IVV7	*Ulmus minor*	Vibo Valentia (RC), Italy	2005	Pepori AL	Bettini et al. (2010)[27]	a, b, c, d, e, f, g
	*Geosmithia* sp. 5	MK971	*Pteleobius vittatus* on *Ulmus minor*	Milovický les, Bulhary, Czech Rep.	2005	Kolařík M	Kolařík et al. (2007)[16]	a, g
	*Geosmithia* sp. 5	MK980	*Pteleobius vittatus* on *Ulmus laevis*	Kančí obora forest, Břeclav, Czech Rep.	2005	Kolařík M	Kolařík et al. (2008)[17]	a, f, g
	*Geosmithia* sp. 5	MK985	Insects species on *Ulmus laevis*	Kančí obora forest, Břeclav, Czech Rep.	2005	Kolařík M	Kolařík et al. (2008)[17]	f
	*Geosmithia* sp. 5	MK542	*Pteleobius vittatus* on *Ulmus* spp.	Bakony Mts., Hungary	2003	Kolařík M	Kolařík et al. (2007)[16]	a, f
	*Geosmithia ulmacea*	CNR23	*Ulmus minor*	Strèdokluky, Czech Rep.	2009	Pepori AL	Pepori et al. (2015)[21]	b, c, f, g
	*Geosmithia ulmacea*	CNR24	*Ulmus minor*	Libick Luh Velky Osek, Czech Rep.	2009	Pepori AL	Pepori et al. (2015)[21]	f, g
	*Geosmithia ulmacea*	MK1515	*Pteleobius vittatus* on *Ulmus minor*	Milovický les, Bulhary, Czech Rep.	2005	Kolařík M	Kolařík et al. (2008)[17]	f
	*Geosmithia ulmacea*	MK924	*Scolytus multistriatus* on *Ulmus minor*	forest near Bulhary, Břeclav, Czech Rep.	2005	Kolařík M	Kolařík et al. (2008)[17]	f
*Geosmithia* from other trees	*Geosmithia fassatiae*	CCF3334	*Quercus pubescens*	Srbsko-Plane, Central Bohemia, Czech Rep.	1993	Kolařík M	Kolařík et al. (2005)	a, b
	*Geosmithia lavendula*	CCF3394	*Chaetopyelius vestitus* on *Pistacia terebinthus*	Dalmatia, Croatia	2003	Kolařík M	Kolařík et al. (2007)[16]	a, b
	*Geosmithia obscura*	CCF3422	*Scolytus intricatus* on *Quercus robur*	North Bohemia, Louny, Czech Rep.	2000	Kolařík M	Kolařík et al. (2005)[15]	a, b
	*Geosmithia putterillii*	CCF3342	*Scolytus rugulosus* on *Prunus* sp.	North Bohemia, Velemin, Czech Rep.	2000	Kolařík M	Kolařík et al. (2004)[14]	a, b

Short-term stock cultures were maintained on malt extract agar (MEA 2%, Oxoid, Basingstoke, UK) at 4 °C and subcultured at 2-week intervals. Long-term stock cultures were maintained on MEA slopes at − 20 °C. Fungal growth rate in dual culture was assessed on MEA and Czapek Dox Agar (CZD, Oxoid, Basingstoke, UK), while mycelial interactions and ONU sporulation were studied in dual cultures growing on elm sapwood agar (ESA) or 2% MEA [29].

### Fungal Growth Rate in Dual Culture (Experiment a)

The reciprocal effect of the presence of *Geosmithia* spp. or *Ophiostoma* spp. on the growth rate of the other species was assessed in dual culture in several trials designed as follows. For each fungal combination, three Petri dishes (90-mm diameter) filled with 20-ml substrate were inoculated by placing two 6-mm diameter mycelial plugs (one of *Geosmithia* spp. and one of *Ophiostoma* spp.), obtained from the edges of actively growing fungal cultures, about 1 cm apart from each other near to the center of dish. Cultures were incubated in the dark at 20 °C and two radii of each colony on the growing edge opposite to the other fungus were measured after 48 h, 3, 5, and 8 days. Three plates per isolate were inoculated with two identical plugs as a control. Daily radial growth rates were compared by one-way ANOVA (Statistica 10, StatSoft Inc.).

Eight *Ophiostoma* spp. isolates (four ONU and four *O. quercus*), 11 species of other Ophiostomatoid fungi, and nine *Geosmithia* spp. isolates (five from elms and four from other trees) were combined in six trials, where *Geosmithia* from elm and from other trees were grown in dual culture with species from the three *Ophiostoma* groups (Table 1). Fungal combinations from the *non-elm system* (oak and conifers) were cultured on both 2% MEA and CZD, while fungal combinations from the *elm system* were grown on 2% MEA.

### Visual Examination of Mycelial Interactions (Experiment b)

In order to determine the existence of a recognition system between Ophiostomatoid fungi and *Geosmithia*, various species of Ophiostomatoid fungi were grown in dual culture with *Geosmithia* spp*.* Inoculations were performed in 90-mm diameter Petri dishes containing 20 ml of substrate (ESA and 2% MEA) as in Experiment a. Three replicates per each fungal combination and medium were prepared and incubated at room temperature in diffuse natural daylight. ESA was used since it had proved very effective for discriminating vegetative compatibility reactions in *O. novo-ulmi* [29, 32], while MEA is a common medium for growing *Geosmithia* spp. Colonies were visually examined after 5 and 10 days for the presence of an antagonism zone or a reaction zone in the region of mycelial contact [32, 33]. Ten *Ophiostoma* spp. isolates (four ONU, four *O. quercus*, and two *O. ulmi*), 11 Ophiostomatoid fungi, and ten *Geosmithia* spp. isolates (six from elms and four from other trees) were combined in dual cultures in eight different trials (*Geosmithia* strains from elm or from other trees were cultivated with fungi from the four *Ophiostoma* groups) (Table 1).

### Observation of Hyphal Interactions in White Light Microscopy (Experiment c)

The mycelial interactions between several *Geosmithia* spp. and strains with different *O. ulmi* and O. *novo-ulmi* strains were studied by white light microscopic observations.

Microscope slides (three per each *Ophiostoma*/*Geosmithia* combination) covered by a water-agar film (2% *w*/*v*) were inoculated with two mycelial plugs (6 mm in diameter) obtained from the edges of actively growing fungal cultures, placed about 1 cm apart from each other. Microscope slides were observed after 2-day incubation (20 °C in the dark) with a Zeiss Axioscop 50 optical microscope equipped with a Nikon digital camera. Images were processed with the Nikon Digital Sight DS-L1 software.

### Transformation of *Geosmithia* sp. 5 “IVV7” with the Green Fluorescent Protein (GFP) Gene (Experiment d)

A GFP-tagged *Geosmithia* strain was obtained to gain a clearer and more detailed vision of the interactions between hyphae of the two fungi. Insertion of the GFP gene into the IVV7 isolate of *Geosmithia* sp. 5 was achieved through *Agrobacterium tumefaciens—*mediated transformation by using strain AGL-1 (kindly provided by Prof. A. Sesma, Universidad Politécnica de Madrid, Spain) containing the pCAMBgfp vector that includes a modified GFP (SGFP) and the hygromycin resistance gene [34]. Transformation was performed according to [34], while stabilization of transformants was carried out as in [35]. Eight independent IVV7-GFP clones were obtained and GFP expression was observed under fluorescence using a Leica MZ FLIII microscope equipped with a mercury lamp and GFP filters (excitation filter at 480/40 and a barrier filter at 510-nm LP). The number of insertions of the pCAMgfp plasmid was determined by southern hybridization using a digoxigenin-labeled GFP probe [27] (not shown). The growth rates of the IVV7-GFP clones and of their parental isolate were determined by inoculating MEA plates with 7-mm diameter mycelial plugs. Plates (at least three per clone) were incubated in the dark at 20 °C and radial growth was measured daily for 12 days. Differences in growth rate were analyzed with the PAST 3× software [36]. Based on southern blot and growth rate, the *Geosmithia*-GFP clone 3.2.2, containing one copy of the GFP gene, was chosen for the experiments.

### Observation of Hyphal Interactions in Fluorescence Microscopy (Experiment e)

The interactions between the hyphae of *O. ulmi* and ONU isolates (ONU ssp. *novo-ulmi* and ONU ssp. *americana*, Table 1) and the transformed *Geosmithia* sp. 5 IVV7-GFP were observed in microscope slides (three replicates for each ONU/*Geosmithia* combination) as described in experiment c. Inoculated slides were incubated in the dark at 20 °C and observed after 2 days under UV light by fluorescence microscopy with a Leica MZ FLIII stereomicroscope (courtesy of Prof. Alessio Mengoni, Department of Biology, University of Florence), equipped with a mercury lamp and GFP filters (excitation filter at 480/40 and barrier filter at 510-nm LP), or white light to verify the autofluorescence of mycoparasite structures. Up to 100 slides per combination were examined.

## Fertility Tests (Experiment f)

The effect of the presence of *Geosmithia* spp. on the production of perithecia in *Ophiostoma* spp. was assessed both in the *elm system* and in the *non-elm* (*oak*) *system*. Petri dishes (90-mm diameter, three replicates per species combination) filled with 20 ml of ESA were inoculated as in experiment a with two mycelial plugs, one from *Geosmithia* spp. and one from *Ophiostoma* spp. mating type A (mtA). Plates were incubated for 12 days in darkness at 20 °C, followed by 7 days in diffuse light. Spores scraped from the surface of an *Ophiostoma* spp. mating type B (mtB) colony that served as a donor strain were applied in 2-cm^2^ patches (five patches per plate) to the plates containing *Ophiostoma* spp. mtA as a recipient strain in combination with *Geosmithia* spp. Plates were incubated for 10 days in diffuse daylight at room temperature. The presence and the number of perithecia (no/cm^2^) were scored under a Nikon SMZ800 stereoscope and data analyzed by means of ANOVA (Statistica 10, StatSoft Inc.). As a control, three plates per species combination were inoculated with only the *Ophiostoma* mtA strain and fertilized with the respective *Ophiostoma* mtB strain. In the *elm system*, 18 *Geosmithia* isolates were combined with two ONU ssp. *novo-ulmi* and ssp. *americana* mtA isolates, respectively, and crossed with the mtB of the corresponding species. In the “*oak system*,” five isolates of *Geosmithia* sp. 5 were tested with 5 *O. quercus* mtA isolates fertilized with a single *O. quercus* mtB isolate (Table 1). Fertility tests were repeated at least three times for each combination.

## Pathogenicity Tests (Experiment g)

The impact of *Geosmithia* in the DED pathosystem was investigated in vivo by means of two pathogenicity tests carried out at the IPSP-CNR experimental nursery (Antella (43° 43′ N 11° 22′ E; 170-m elevation, Florence, Italy). Several *Geosmithia* spp. and ONU strains were inoculated alone and in combination in the elm clone *Ulmus* “Commelin,” which was chosen for being extremely susceptible to DED [37]. Hundred five-year-old saplings growing in rows (spacing 0.5 m within × 1 m between rows) in a substrate comprising commercial loam to a depth of 2-m drip irrigated were inoculated. The bed was cleared and plowed prior to planting and weeded monthly thereafter. Two pathogenicity tests were performed as follows:

1. In May 2013, *Ulmus* Commelin (six individuals per fungal strain) was inoculated with each of seven *Geosmithia* spp. strains with a single wound per plant in the upper third of the main stem. Inoculations were performed following the protocol established by Santini et al. [38] for ONU inoculations, i.e., by cutting through the bark to the younger sapwood with a knife blade bearing two 0.2-ml drops of a 1 × 10^6^/ml fungal spore suspension so that the inoculum was absorbed in the sap flux.
2. In May 2014, 12 *Ulmus* Commelin individuals were co-inoculated with the same technique as above with a spore suspension containing *Geosmithia* sp. 5 (IVV7) and ONU ssp. *novo-ulmi* “H328.” The concentration of each fungus in the inoculum was adjusted to 1 × 10^6^ spores/ml. As a control, 12 trees were inoculated with only *Geosmithia* sp. 5 (IVV7) and 12 trees with only ONU ssp. *novo-ulmi* H328. *Geosmithia* sp. 5 was chosen for the experiment because it is one of the most common species on elm, and IVV7 is our model strain for this species [21, 22, 39]. *O. novo-ulmi* ssp. *novo-ulmi* H328 is a well-known and very aggressive strain [38, 40]. Symptoms of disease were observed at 4 weeks (percentage defoliation) and 12 months (percentage of crown dieback) after inoculation by three independent assessors. Pathogenicity data were analyzed by means of ANOVA (Statistica 10, StatSoft Inc.). Arcsine transformation was applied before statistical analyses to correct percentage data for departure from normality assumption.

## Results

### Fungal Growth Rate in Dual Culture (Experiment a)

#### Elm System

**Fig. 1:**
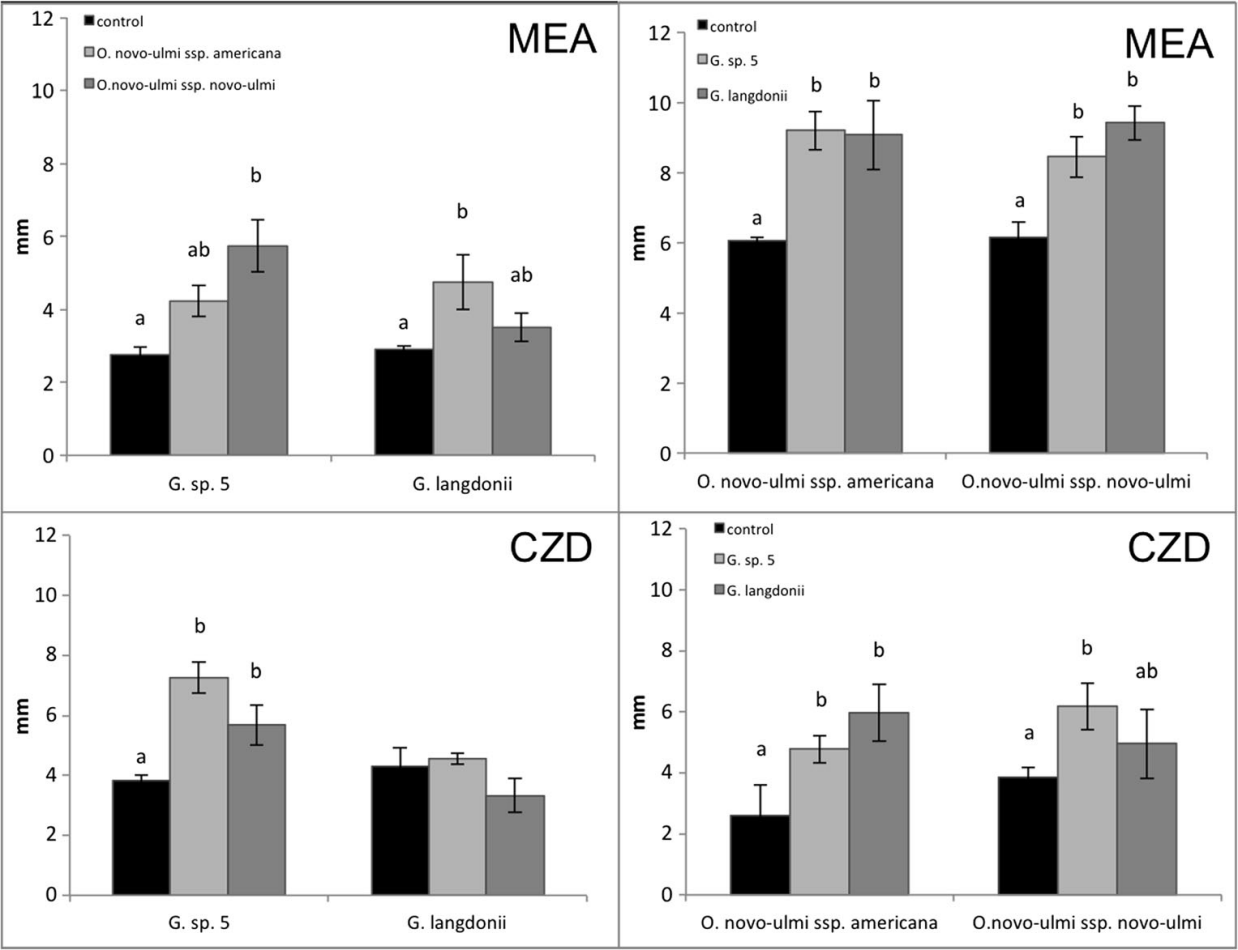
Fungal growth rate in dual culture. Left, growth rate of *Geosmithia* spp. with *Ophiostoma novo-ulmi* ssp. *novo-ulmi* and ssp. *americana* on MEA (2%) and CZD; right, growth rate of *O. novo-ulmi* ssp. *novo-ulmi* and ssp. *americana* with *Geosmithia* spp. on MEA (2%) and CZD. Values sharing the same letters are not significantly different based on Duncan’s test (*p* ≤ 0.05)

The growth rate of ONU strains was generally higher in dual culture with *Geosmithia* spp. isolated from elm than in pure culture, both on MEA and CZD (Fig. 1). In the same trial, the growth rate of *Geosmithia* did not show such a clear and consistent trend. Within each species, all strains grew at the same rate (non-significant Duncan test, *p* > 0.05); therefore, different strains were used as replicates in subsequent analyses.

#### Non-elm Systems

Both in the *oak system* (*O. quercus* in dual culture with *Geosmithia* from elm, oak or other trees) and in the *conifers system* (Ophiostomatoid fungi from conifers in dual culture with *Geosmithia* from elm, conifers, or other trees), the mean radial growth in dual culture was unchanged compared to controls in all tested fungi (results not shown).

### Visual Examination of Mycelial Interactions (Experiment b)

**Fig. 2:**
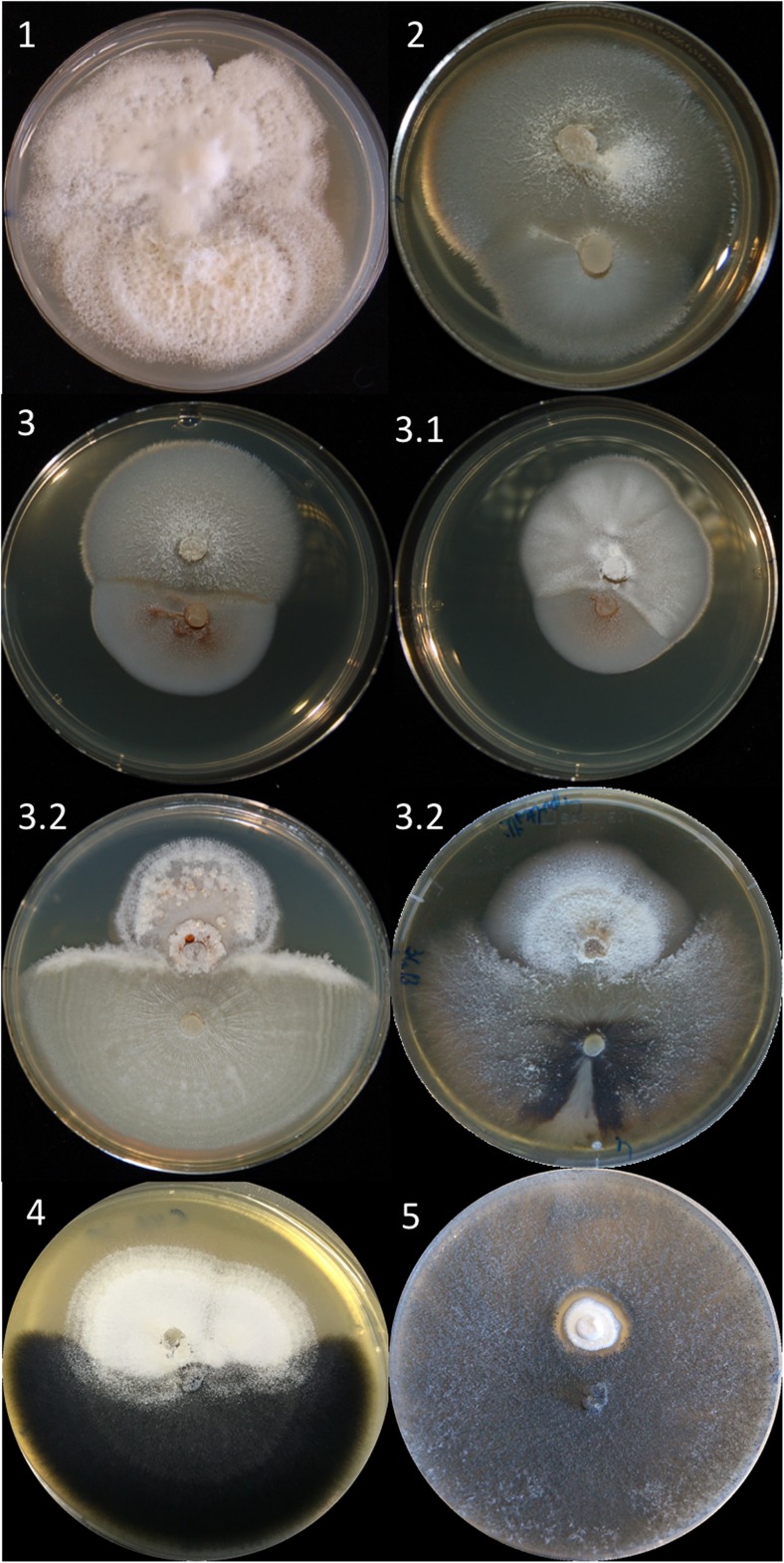
Type of mycelial interactions on MEA (2%) (the mycelium at the figure’s top belongs to *Geosmithia* spp. in all cases): type 1, *G. omnicola* (CNR8)-*Ophiostoma novo-ulmi* ssp. *novo-ulmi* (H327); type 2, *G. flava* (CNR120)-*Ceratocystis minuta* (KW/3/4); type 3, *G. flava* (CNR120)-*Rhexographium fimbriasporum* (R/4/1/2); type 3.1, *G. omnicola* (CNR8)-*Rhexographium fimbriasporum* (R/4/1/2); type 3.2, *G. obscura* (CCF3422)-*O. ulmi* (E2); type 3.2, *G. putterillii* (CCF3342)-*Ophiostoma ainoae* (KW/Ku/29); type 4, *G. flava* (CNR120)-*Ophiostoma clavatum* (AC/1/1/1); type 5, *G. ulmacea* (CNR23)-*Endoconidiophora polonica* (KOW/Ku/41). The different types of mycelial interaction are described in the “Results” section (experiment b)

The reactions observed between the mycelia of Ophiostomatoid fungi and *Geosmithia* species were here classified into five main types, ranging from fully intermingling colonies to mutual growth inhibition (Table 2, Fig. 2):

Type 1, *fully intermingling*: complete equal bidirectional mycelial penetration. After 10 days, the two colonies were not distinguishable. Neither boundaries nor changes in color were recognizable in the mycelium.
Type 2, *intermingling*: the two colonies were easily recognizable, but no barrage line was visible and hyphae were intermingled along the junction line.
Type 3, *mutual incompatibility*: a diffuse mycelial barrage, 1 to 2 mm large, was clearly visible along the junction line between the two colonies;
  3.1: diffuse mycelial barrage developed by *Geosmithia* spp. No visible barrage was produced by Ophiostomatoid fungi.
  3.2: diffuse mycelial barrage developed by Ophiostomatoids. No visible barrage was produced by *Geosmithia* spp.
Type 4, *strong growth inhibition and overgrowth*: the growth of Ophiostomatoid fungi was inhibited at a distance of about 1–2 mm from *Geosmithia* hyphae, which later occupied the gap spreading eventually over the mycelium of the co-cultured Ophiostomatoid species.
Type 5, *mutual inhibition*: a 1–5-mm wide demarcation zone, where the aerial mycelium was missing, was visible along the confrontation line.

**Table 2:** Reaction patterns between paired *Geosmithia* spp. and Ophiostomatoid fungi on 2% MEA. Key: type 1, fully intermingling: equal bidirectional penetration; type 2, intermingling: colonies intermingling along junction line; type 3, mutual incompatibility: barrages along junction gap; type 3.1, diffuse mycelial barrages developed by *Geosmithia* spp.; type 3.2, diffuse mycelial barrages developed by Ophiostomatoid fungi; type 4, strong growth inhibition and overgrowth: inhibition of Ophiostomatoid fungi growth by *Geosmithia*; type 5, mutual inhibition: inhibition zone; nt, not tested

Species	Isolate number	*G. langdonii* MK1643	*G. flava* CNR120	*G. ulmacea* CNR23	*G. omnicola* CNR8	*G.* sp. 5 IVV7	*G.* sp. 20 CNR132	*G. obscura* CCF3422	*G. putterillii* CCF3342	*G. lavendula* CCF3394	*G. fassatiae* CCF3334
*O. novo-ulmi* ssp. *novo-ulmi* mtA	H327	1	1	1	1	1	1	4	4	4	4
*O. novo-ulmi* ssp. *novo-ulmi* mtB	H328	1	1	1	1	1	1	4	4	4	4
*O. novo-ulmi* ssp. *americana* mtA	H172	1	1	1	1	1	1	4	4	4	5
*O. novo-ulmi* ssp. *americana* mtB	H363	1	1	1	1	1	1	4	4	3	5
*O. ulmi* mtA	R21	4	3	5	3	3	3	3	3	3	3
*O. ulmi* mtB	E2	4	3	2	4	5	3	3	3	3	4
*O. quercus* mtA	CTK2-s	3	1	1	1	3.2	1	4	2	4	4
*O. quercus* mtA	CTK120-s	3	3	2	3	3.2	3	3	5	4	3
*O. quercus* mtB	RZ/7-s	3	5	3	3	3.2	3	3	3	3.2	3
*O. quercus* mtA	TB/35-s	3	3	3	3	3.2	3	3	4	4	3
*O.* cf. *picea*	AT30-s	3	nt	nt	nt	1	nt	4	5	3.1	5
*O. kryptum*	Hasd/3	4	2	2	3.2	1	2	2	4	4	5
*O.* cf. *clavatum*	AC/1/1/1	3.2	4	4	4	1	4	4	4	4	4
*Ceratocystis polonica*	KOW/Ku/41	4	3	5	1	1	2	1	4	5	4
*Ceratocystis* cf. *minuta*	KW/3/4	2	2	5	4	1	1	3.1	4	2	4
*Leptographium sp.1*	KW/2/2/2/1	3.2	4	2	4	3	4	3.1	4	4	3.2
*O. ainoae*	KW/Ku/29	3.2	nt	nt	nt	2	nt	3.1	3.2	4	3.2
*O. tetropii*	CBS428.94	4	nt	nt	nt	1	nt	3.1	3.2	3.2	3.2
*Graphium fimbrisporum*	R/4/1/2	3	3	5	3.1	2	1	3	3.2	3	3.2
*Grosmannia piceiperda*	KW/4/2/2/1	4	4	1	3.2	2	1	1	1	1	1
*Grosmannia penicillata*	KW/4/2/6/2	4	4	4	4	2	1	4	2	4	4

A fully intermingling reaction (type 1) was observed every time that two species from the *elm system* were grown in dual culture. Interactions between species from *non-elm systems* were generally characterized, with few exceptions, by various signs of mycelial inhibition, from a barrage to a wide gap along the junction line (types 3–5), revealing a recognition system between the two fungi. Compatible reactions of types 1 and 2 were observed only in dual cultures of some Ophiostomatoid fungi with *Geosmithia* sp. 5 IVV7 (Fig. 2).

### Mycoparasitic Interactions Between *Geosmithia* from Elm and *Ophiostoma* in White and Fluorescent Light Microscopy (Experiments c, e)

Under the white light microscope, the mycelia of wild type strains of *Geosmithia* spp. from elm and of *O. ulmi* or ONU cultured together appeared to grow towards each other, with profuse hyphal growth and production of mycelial tufts (Online Resource 1). Signs of mycoparasitism by *Geosmithia* on *Ophiostoma* hyphae, such as the formation of coilings, appressoria-like branches, pseudopod-like structures, or short hooks, were common (Online Resource 1).

**Fig. 3:**
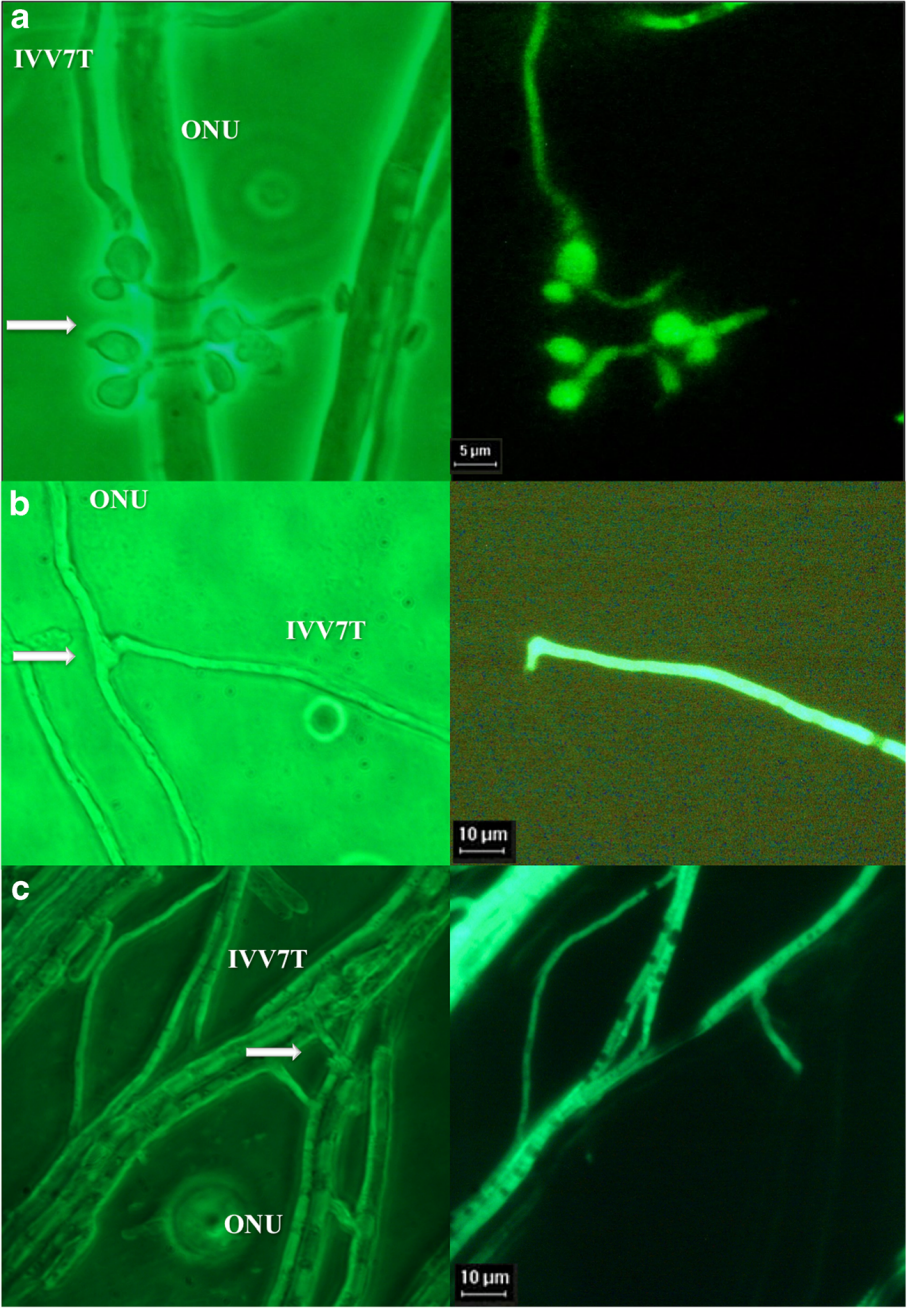
**a**–**c** Interacting hyphae of *Ophiostoma novo-ulmi* (ONU) and *Geosmithia* sp. 5 “IVV7”-GFP (IVV7T). On the left both species are observed under white light, while on the right only *Geosmithia* hyphae are visible in UV light (under UV light a green specific signal is due to the GFP transformation). Arrows indicate possible parasitic structures formed by *Geosmithia*

In the *elm system*, the formation by *Geosmithia* on *Ophiostoma* hyphae of structures that are typically observed during mycoparasitic attack was confirmed with increased evidence when the *Geosmithia* sp. 5 IVV7-GFP clone 3.2.2 was observed in dual culture with both *O. ulmi* (not shown) and ONU (Fig. 3a–c) under UV light.

### Fertility Test (Experiment f)

**Fig. 4:**
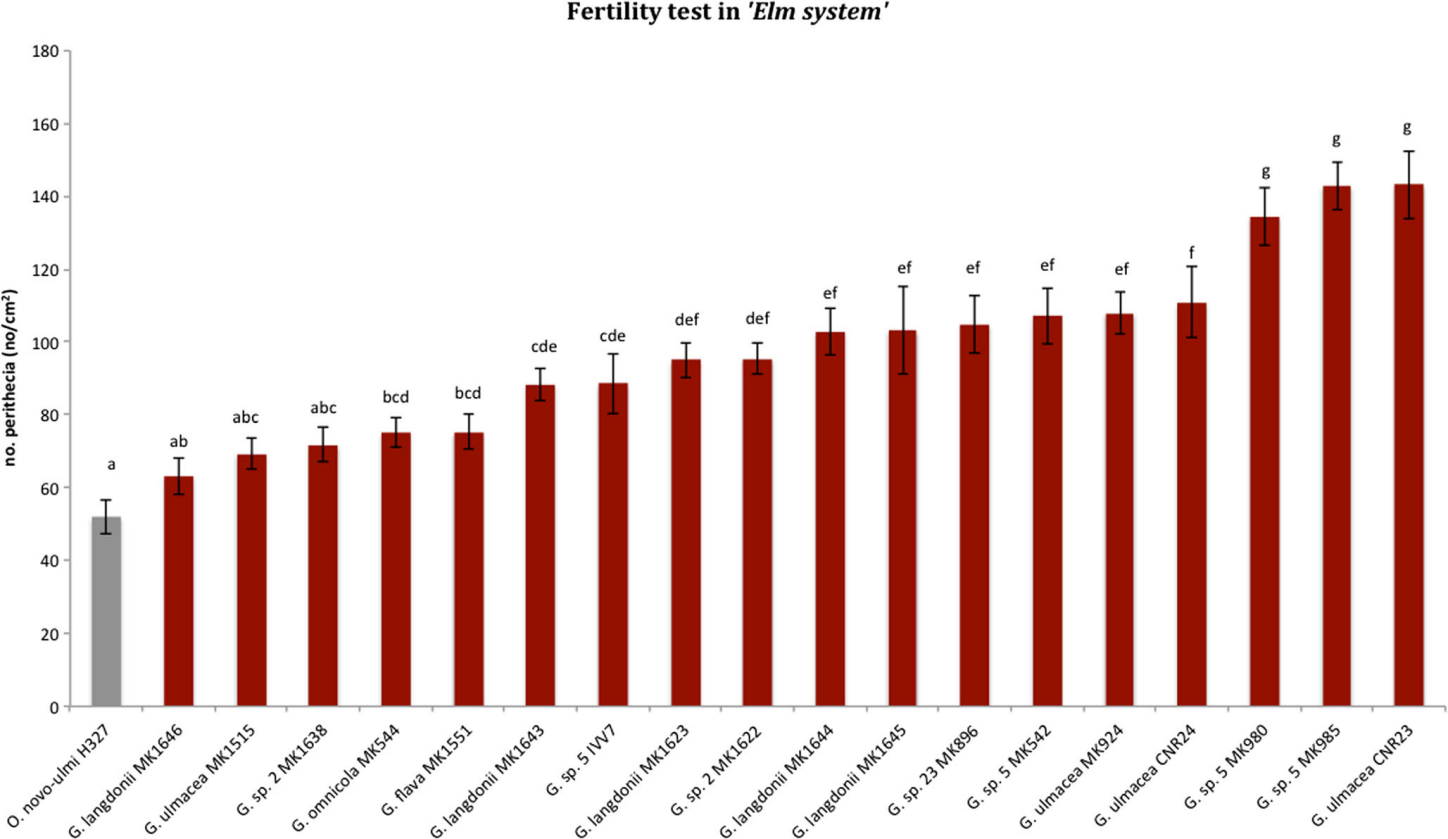
Fertility test between species from the “*elm system.*” The number of perithecia formed by *Ophiostoma* spp. in dual-culture with *Geosmithia* spp. is shown in red, while gray bars correspond to the control. Duncan’s test was applied to test for differences in means. Values sharing the same letters are not significantly different (*p* ≤ 0.05)

In the *elm system*, ONU ssp. *novo-ulmi* mtA (H327) fertilized by a mtB strain (H328) produced a significantly higher number of perithecia (Duncan test, *p* < 0.05) in dual cultures with *Geosmithia* spp. isolates than in control crosses where *Geosmithia* was absent (Fig. 4). On the contrary, ONU ssp. *americana* cultivated with *Geosmithia* spp. did not produce perithecia after fertilization with the opposite mating type.

In the *oak system* instead, all strains of *O. quercus* mtA fertilized with opposite mtB isolates produced an equal number of perithecia whether or not they were grown in dual culture with *Geosmithia* sp. 5 (results not shown).

### Pathogenicity Tests (Experiment g)

The addition of spores of *Geosmithia* species to the suspension of ONU spores used for artificial inoculations reduced DED symptoms, both defoliation (4 weeks after inoculation, Duncan test *p* ≤ 0.05) and dieback (12 months after inoculation, Duncan test *p* ≤ 0.05), in the elm clone *Ulmus* Commelin compared to controls inoculated with ONU alone (Table 3). While inoculation with ONU produced severe DED symptoms, elms remained substantially asymptomatic after inoculation with only *Geosmithia* (Table 3), regardless of the *Geosmithia* species applied (results not shown).

**Table 3:** Pathogenicity test. Mean disease scores not sharing a common letter differ significantly by Duncan’s test (*p* < 005). *N* number of tested isolates, mean % mean percentage of symptoms, SE standard error

	*N*	4 weeks			12 months		
		Mean defoliation %	SE		Mean dieback%	SE	
*O. novo-ulmi*	20	24.210	3.109	a	55.518	7.255	a
*O. novo-ulmi* + *Geosmithia* sp. 5	20	18.329	2.330	b	17.658	6.622	b
*Geosmithia* sp. 5	60	2.014	0.854	c	1.084	0.577	b

In particular, 12 months after inoculation, when the plant reaction is stable and can be considered as conclusive, dieback was much more severe in the plants inoculated with ONU alone than in the plants co-inoculated with ONU and *Geosmithia* spp. (55.5 vs. 17.6%, respectively). *O. novo-ulmi* was always successfully re-isolated from xylem of inoculated trees, while none of the *Geosmithia* species used was re-isolated.

## Discussion

Millions of elms vanished from Europe and North America over the last 100 years because the alien fungi responsible for DED established in the areas of introduction a new association with native EBBs that became extremely efficient vectors of the disease. The same beetles also have a high-fidelity association with fungi of the genus *Geosmithia* [16].

Geosmithias are generally considered as saprotrophs or endophytes [17]. However, in elms, they have never been isolated from dead wood or from healthy trees, but they were only found in beetles’ galleries [21]. High frequency HGT of the cerato-ulmin (*cu*) gene between ONU and *Geosmithia* spp. [22] suggests that between the two species exists a relationship that goes beyond simple sharing of habitat and vectors and is much closer.

The present study provides direct and indirect evidence of mycoparasitism on ONU by many *Geosmithia* isolates specific to the *elm system*. Should this be the case, it might be concluded that the transfer of the *cu* gene observed between ONU and *Geosmithia* may be described as prey-derived HGT. The integration into the genome of sequences derived from organisms consumed as food has frequently been reported, leading to the “you are what you eat” hypothesis [41], both in phagotrophic eukaryotes harboring genes from food sources [42, 43] and in prokaryotes such as the bacteriolytic *Bdellovibrio bacteriovorus* HD100 [44, 45].

The higher growth rate observed in ONU in dual culture with *Geosmithia* might be regarded as a sort of “escape in space” (sensu Janzen) [46] of ONU from *Geosmithia* towards an area free from the “enemy.” The absence of mycelial interaction between *Geosmithia* and ONU is consistent with the hypothesis that the two organisms represent a newly formed host-pathogen system. In the *oak system* and in the *conifers system*, recognition between *Geosmithia* spp. and Ophiostomatoid fungi was the norm, with very few exceptions. A weak intermingling reaction with no mycelial barrages along the junction line was observed in dual culture (1) between *Geosmithia* sp. 5 IVV7 and many *Ophiostomatales*, (2) in all the combinations of *Geosmithia* spp. with Ophiostomatoid fungi in *non-elm systems*, and (3) when *Geosmithia* sp. 20 was co-cultured with *Leptographium piceaperdum*, the most common *Ophiostomatales* species associated with spruce beetles [47]. In no instance, however, structures typical of parasitic behavior were formed in these combinations.

Therefore, *Geosmithia* parasitic behavior seems to be specific to the *elm system*. In fact, in most of the *non-elm systems* challenged here, similar interactions were not observed. If this hypothesis proves true, then IVV7 is the isolate displaying the most evident mycoparasitic behavior. This behavior explains its ability to overtake the host defense mechanisms and to grow over it. In this system, ONU represents a widely available carbon source exploitable by *Geosmithia* fungi.

Comparing the present results with what is known for *Trichoderma*, a fungal genus well known as a mycoparasite and biocontrol agent [48–51], several similarities can be identified. *Trichoderma* attraction to and growth towards its host seems to be stimulated at a distance by the recognition of diffusible signals, such as oligochitins [52]. Mycoparasitism in *Trichoderma* spp. involves hydrophobins and hydrophobin-like proteins, such as cerato-platanins. Class II hydrophobins HYTLO1 and TvHydII1, isolated respectively from *Trichoderma longibrachiatum* MK1 [53] and *T. viride* [54], are required for mycoparasitic activity against phytopathogenic fungi to grow over their hosts. *Trichoderma* *harzianum* cerato-platanin Epl-1 [50, 55] also has key functions in the mycoparasitic process, as a self-recognition factor or by modulating hyphal coiling and mycoparasitism-related gene expression, and in the interaction with the host plant [55].

Similarly, in the *Geosmithia*-ONU system, the attraction signal seems to act at a distance without physical contact. Upon contact, *Geosmithia* hyphae coil around or grow along ONU hyphae, forming appressoria-like structures that may be used for penetrating ONU (Fig. 4). *Geosmithia* fungi produce a class II hydrophobin, GEO1, which could be involved in the attachment to other hydrophobic structures, e.g., insect exoskeleton and hyphae of other fungi [25, 56]. The mode of action and the mechanisms involved in the *Geosmithia*-ONU-elm interaction are still unknown, but GEO1 might play a similar role as *Trichoderma* hydrophobins and Epl-1, promoting mycoparasitic activity and inducing local and systemic defenses in plants [53–55].

Brasier [57–59] showed that *Trichoderma* could trigger sexual reproduction in many isolates of the *Phytophthora* A2 compatibility group by producing volatile antibiotics, an effect which is more likely a defense mechanism specifically evolved in *Phythopthora* than an incidental phenomenon. In the present study, *Geosmithia* spp. tested in fertility trials showed on ONU a similar effect as *Trichoderma* spp. on *Phytophthora*. Within the *elm system*, *Geosmithia* was shown to induce a significantly higher production of proto-perithecia in all isolates of ONU mtA and of perithecia when fertilized by the opposite mtB. A possible interpretation is that *Geosmithia* (predator) stimulates in ONU (prey) the “escape from the predator in time” [46] reaction, possibly increasing the evolutionary potential of ONU populations by boosting sexual reproduction and recombination. Such an effect was not observed in the *oak system*.

Artificial inoculation with ONU resulted in typical symptoms of DED in elms, while no sign of disease was observed when *Geosmithia* alone was inoculated. In the case of co-infection, the presence of *Geosmithia* reduces DED symptoms. This could be attributed either to its mycoparasitic activity or to the enhancement of defense mechanisms in elm. A similar effect is well known in *Trichoderma* fungi that not only protect plants directly by killing other fungi and nematodes but also induce resistance against plant pathogens [51]. Based on these results, *Geosmithia* is not a pathogen on elm, in contrast with the observation by Hänzi et al. [60]. In no case, we were able to re-isolate *Geosmithia* from artificially infected elms, nor was it reported among the endophytic cohort of saprotrophs of elm trees [61]. The amount of the fungus in elm tissues could be too low to be detected with standard techniques and require a more sensitive method such as a specific qPCR assay. It could as well be moved to a district of the tree different from the xylem.

If mycotrophy towards many plant pathogenic fungi has long been the original lifestyle of *Trichoderma*, in *Geosmithia*, it appears to be a recent event. The DED epidemics that occurred in Europe during the past century created the conditions for *Geosmithia* development, reproduction, and dissemination by increasing the number of suitable habitats for both ONU and *Geosmithia* spp. These conditions may have favored the discovery and systematic study of the genus *Geosmithia* by the scientific community [13]. This hypothesis is supported by the finding that the *cu* gene was transferred to *Geosmithia* from ONU, but not from *O. ulmi* [22]. As the appearance of ONU in Europe can be dated at around the 1960s [2], HGT between the two fungi should be a very recent and currently ongoing event in Europe. The lack of recognition between *Geosmithia* and ONU in the *elm system* confirms that they were geographically isolated and interacted only recently. The *cu* gene was not found in any of the *Geosmithia* isolates obtained from the *non-elm system*.

A DED epidemic outbreak is governed by the population dynamics of the host, the pathogen and its vector, and also by the rate of sexual reproduction of the pathogen, which can influence the risk of fungus viral disease outcome and, lastly, by the presence of mycoparasitic fungi as *Geosmithia* species [22].

The system can be described as a classical Lotka-Volterra model in which the predator, ONU, supported by beetles as vectors, consumes the prey, leading to depletion of elm population and, consequently, of both the predator and the vector populations. When the predator population is low, the prey is able to thrive, thereby putting the ecosystem through cycles of “boom-and-bust.” In the long run, the intervention of new factors may lead to stabilization of the population dynamics. Many polyphagous organisms are able to switch to different carbon sources over time in response to variation in the local ecosystem. Therefore, as ONU became more and more abundant in the community (getting in contact more frequently with organisms sharing the same habitat and vectors), we expect that another organism, even mildly pathogenic as *Geosmithia*, might have adapted to attack this new host species and reproduce on it, which would lead to an increased degree of parasitism [62].

In the early 1980s, many researchers focused on possible agents of biological control of DED as bacteria [47, 63–70]. Unfortunately, none of these authors could provide evidence that any of these microorganisms might become a successful and widespread competitor or parasite of DED fungi. The main reasons for these drawbacks are that these antagonistic species either are limited by environmental factors [69] or have no vectors able to spread them. On the contrary, *Geosmithia* species benefit from a widespread distribution and a strict association with effective insect vectors.

Here, it was shown that *Geosmithia* is an important element in the DED network, making it even more complex, yet probably less detrimental for elms, and more stable over time. There is increasing evidence that the health or disease status of a given organism is not just the result of the interaction between host and pathogen but depends on a complex interaction between each partner and its microbial community (holobiont), which in the end determines the outcome of the infection. Therefore, the fate of the infected elm is not determined only by ONU, but it rather depends on the DED network which may be defined as a holobiont, i.e., the totality of all beings involved comprising ONU, d-factor viruses, EBBs, mites, and also *Geosmithia*.

Moreover, as Geosmithias living in the *elm system* are able to mycoparasitize ONU and to reduce DED symptoms in artificially inoculated plants, these fungi might be used as biocontrol agents against ONU. Further research is certainly needed to assess the mechanisms that allow Geosmithias, when co-inoculated with ONU, to attenuate DED symptoms, and to define both how to exploit this effect and how to artificially spread “elm Geosmithias.” However, such a holistic approach would reinforce the conviction that a different management of diseases in natural ecosystems is possible.

## Electronic supplementary material

ESM 1 (PDF 1728 kb)

ESM 2 (DOCX 75 kb)

## References

[1] Six DL (2013). The bark beetle holobiont: why microbes matter. J. Chem. Ecol..

[2] Brasier CM (1991). *Ophiostoma novo-ulmi* sp. nov., causative agent of current Dutch elm disease pandemics. Mycopathologia.

[3] Fransen JJ (1939). Iepenziekte Iepenspintkevers an beider bestrijding [elm disease, elm beetles and their control].

[4] Santini A, Faccoli M (2015). Dutch elm disease and elm bark beetles: a century of association. iForest.

[5] Moser JC, Konrad H, Blomquist SR, Kirisits T (2010). Do mites phoretic on elm bark beetles contribute to the transmission of Dutch elm disease?. Naturwissenschaften.

[6] Brasier CM (1983). A cytoplasmically transmitted disease of *Ceratocystis ulmi*. Nature.

[7] Webber JF (1987). Influence of the d2 factor on survival and infection by the Dutch elm disease pathogen *Ophiostoma ulmi*. Plant Pathol..

[8] Brasier CM, Buck KW (1986). The d-factor in *Ceratocystis ulmi*—its biological characteristics and implications for Dutch elm disease. Fungal Virology.

[9] Brasier CM, Dunn CP (2000). Viruses as biological control agents of the Dutch elm disease fungus *Ophiostoma novo-ulmi*. The elms. Breeding, conservation, and disease management.

[10] Paoletti M, Buck KW, Brasier CM (2006). Selective acquisition of novel mating type and vegetative incompatibility genes via interspecies gene transfer in the globally invading eukaryote *Ophiostoma novo-ulmi*. Mol. Ecol..

[11] Buck KW, Brasier CM, Tavantzis SM (2002). Viruses of the Dutch elm disease fungi. dsRNA genetic elements: concepts and applications in agriculture, forestry and medicine.

[12] Brasier CM, Kirk SA (2010). Rapid emergence of hybrids between the two subspecies of *Ophiostoma novo-ulmi* with a high level of pathogenic fitness. Plant Pathol..

[13] Pitt JI (1979). *Geosmithia* gen. nov. for *Penicillium lavendulum* and related species. Can. J. Bot..

[14] Kolařík M, Kubátová A, Pažoutová S, Šrůtka P (2004). Morphological and molecular characterisation of *Geosmithia putterillii*, *G. pallida* comb. nov. and *G. flava* sp. nov., associated with subcorticolous insects. Mycol. Res..

[15] Kolaøík M, Kubátová A, Cepicka I, Pažoutová S, Šrùtka P (2005) A complex of three new whitespored, sympatric, and host range limited Geosmithia species. Mycol Res 109:1323–1336. 10.1017/S0953756205003965 16353633

[16] Kolařík M, Kostovčík M, Pažoutová S (2007). Host range and diversity of the genus *Geosmithia* (Ascomycota: Hypocreales) living in association with bark beetles in the Mediterranean area. Mycol. Res..

[17] Kolařík M, Kubátová A, Hulcr J, Pažoutová S (2008). *Geosmithia* fungi are highly diverse and consistent bark beetle associates: evidence from their community structure in temperate Europe. Microb. Ecol..

[18] Kolařík M, Freeland M, Utlet C, Tisserat N (2011). *Geosmithia morbida* sp. nov., a new phytopathogenic species living in symbiosis with the walnut twig beetle (*Pityophthorus juglandis*) on Juglans in USA. Mycologia.

[19] Kolařík M, Jankowiak R (2013). Vector affinity and diversity of *Geosmithia* fungi living on subcortical insects inhabiting Pinaceae species in Central and Northeastern Europe. Microb. Ecol..

[20] McPherson BA, Erbilgin N, Bonello P, Wood DL (2013). Fungal species assemblages associated with *Phytophthora ramorum*-infected coast live oaks following bark and ambrosia beetle colonization in northern California. Forest Ecol Manag.

[21] Pepori AL, Kolařík M, Bettini PP, Vettraino AM, Santini A (2015). Morphological and molecular characterisation of *Geosmithia* species on European elms. Fungal Biol.

[22] Bettini PP, Frascella A, Kolařík M, Comparini C, Pepori AL, Santini A, Scala F, Scala A (2014). Widespread horizontal transfer of the cerato-ulmin gene between *Ophiostoma novo-ulmi* and *Geosmithia* species. Fungal Biol.

[23] Scala F, Bertelli E, Coppola L, Del Sorbo G, Tegli S, Scala A (1997). Comparative determination of *cerato-ulmin* on cell surface and in mycelial extracts of pathogenic and non-pathogenic *Ophiostoma* species. Mycol. Res..

[24] Carresi L, Comparini C, Bettini PP, Pazzagli L, Cappugi G, Scala F, Scala A (2008). Isolation of the orthologue of the cerato-ulmin gene in *Ophiostoma quercus* and characterization of the purified protein. Mycol. Res..

[25] Temple B, Horgen PA (2000). Biological roles for cerato-ulmin, a hydrophobin secreted by the elm pathogens, *Ophiostoma ulmi* and *O. novo-ulmi*. Mycologia.

[26] Cizková D, Sřůtka P, Kolařík M, Kubátová A, Pažoutová S (2005). Assessing the pathogenic effect of *Fusarium*, *Geosmithia* and *Ophiostoma* fungi from broad-leaved trees. Folia Microbiol.

[27] Bettini PP, Baraldi R, Rapparini F, Melani L, Mauro ML, Bindi D, Buiatti M (2010). The insertion of the *Agrobacterium rhizogenes rolC* gene in tomato (*Solanum lycopersicum* L.) affects plant architecture and endogenous auxin and abscisic acid levels. Sci Hort.

[28] Wingfield MJ, Barnes I, de Beer ZW, Roux J, Wingfield BD, Taerum SJ (2017) Novel associations between ophiostomatoid fungi, insects and tree hosts: current status-future prospects. Biol. Invasions. 10.1007/s10530-017-1468-3

[29] Brasier CM, Stipes RJ, Campana RJ (1981). Laboratory investigation of *Ceratocystis ulmi*. Compendium of elm diseases.

[30] Pipe ND, Buck KW, Brasier CM (1995) Molecular relationships between Ophiostoma ulmi and the NAN and EAN races of O. novo-ulmi determined by RAPD markers, Mycol Res 99:653–658. 10.1016/S0953-7562(09)80522-4

[31] Gibbs, J. N., Brasier, C. M., McNabb, H. S. and Heybroek, H. M. (1975), Further studies on pathogenicity in Ceratocystis ulmi. Eur J For Pathol 5:161–174. 10.1111/j.1439-0329.1975.tb00461.x

[32] Brasier CM, Jennings DH, Rayner ADM (1984). Inter-mycelial recognition systems in *Ceratocystis ulmi*: their physiological properties and ecological importance. The ecology and physiology of the fungal mycelium.

[33] Punja ZK, Grogan RG (1983). Hyphal interactions and antagonism among field isolates and single-basidiospore strains of *Athelia* (*Sclerotium*) *rolfsii*. Phytopathology.

[34] Sesma A, Osbourn AE (2004). The rice leaf blast pathogen undergoes developmental processes typical of root-infecting fungi. Nature.

[35] Sarrocco S, Falaschi N, Vergara M, Nicoletti F, Vannacci G (2007). Use of *Fusarium oxysporum* f. sp. *dianthi* transformed with marker genes to follow colonization of carnation roots. J. Plant Pathol..

[36] Hammer Ø, Harper DAT, Ryan PD (2001). PAST: paleontological statistics software package for education and data analysis. Palaeontol. Electron..

[37] Heybroek HM, Sticklen MB, Sherald JL (1993). The Dutch elm breeding program. Dutch elm disease research.

[38] Santini A, Fagnani A, Ferrini F, Ghelardini L, Mittempergher L (2005). Variation among Italian and French elm clones in their response to *Ophiostoma novo-ulmi* inoculation. Forest Pathol.

[39] Scala A, Comparini C, Tegli S, Scala F (2007). A non-*Ophiostoma* fungus expresses the gene encoding the hydrophobin cerato-ulmin. J. Plant Pathol..

[40] Brasier CM (1986). Comparison of pathogenicity and cultural characteristics in the EAN and NAN aggressive subgroups of *Ophiostoma ulmi*. T Brit Mycol Soc.

[41] Doolittle WF (1998). You are what you eat: a gene transfer ratchet could account for bacterial genes in eukaryotic genomes. Trends Genet..

[42] Loftus BJ, Fung E, Roncaglia P, Rowley D, Amedeo P, Bruno D, Allen JE (2005). The genome of the basidiomycetous yeast and human pathogen *Cryptococcus neoformans*. Science.

[43] Yue J, Hu X, Huang J (2013). Horizontal gene transfer in the innovation and adaptation of land plants. Plant Signal. Behav..

[44] Rendulic S, Jagtap P, Rosinus A, Eppinger M, Baar C, Lanz C, Keller H, Lambert C, Evans KJ, Goesmann A, Meyer F, Sockett RE, Schuster SC (2004). A predator unmasked: life cycle of *Bdellovibrio bacteriovorus*. Science.

[45] Gophna U, Charlebois RL, Doolittle WF (2006). Ancient lateral gene transfer in the evolution of *Bdellovibrio bacteriovorus*. Trends Microbiol..

[46] Janzen DH (1971). Escape of *Cassia grandis* L. beans from predators in time and space. Ecology.

[47] Jankowiak R, Kacprzyk M, Młynarczyk M (2009). Diversity of ophiostomatoid fungi associated with bark beetles (Coleoptera: Scolytidae) colonizing branches of Norway spruce (*Picea abies*) in southern Poland. Biologia.

[48] Viterbo A, Horwitz BA, Borkovich K, Ebbole DJ (2010). Mycoparasitism. Cellular and molecular biology of filamentous fungi.

[49] Matarese F, Sarrocco S, Gruber S, Seidl-Seiboth V, Vannacci G (2012). Biocontrol of *Fusarium* head blight: interactions between *Trichoderma* and mycotoxigenic *Fusarium*. Microbiology.

[50] Mukherjee PK, Horwitz BA, Herrera-Estrella A, Schmoll M, Kenerley CM (2013). *Trichoderma* research in the genome era. Annu. Rev. Phytopathol..

[51] Lorito M, Woo SL, Lugtenberg B (2015). *Trichoderma*: a multi-purpose tool for integrated pest management. Principles of plant-microbe interactions. Microbes for sustainable agriculture.

[52] Cortes C, Gutierrez A, Olmedo V, Inbar J, Chet I, Herrera-Estrella A (1998). The expression of genes involved in parasitism by *Trichoderma harzianum* is triggered by a diffusible factor. Mol Gen Genet.

[53] Ruocco M, Lanzuise S, Lombardi N, Woo SL, Vinale F, Marra R, Varlese R, Manganiello G, Pascale A, Scala V, Turrà D, Scala F, Lorito M (2015). Multiple roles and effects of a novel *Trichoderma* hydrophobin. Mol. Plant-Microbe Interact..

[54] Guzmán-Guzmán P, Alemán-Duarte MI, Delaye L, Herrera-Estrella A, Olmedo-Monfil V (2017). Identification of effector-like proteins in *Trichoderma* spp. and role of a hydrophobin in the plant-fungus interaction and mycoparasitism. BMC Genet..

[55] Gomes EV, do Nascimento Costa M, de Paula RG, Ricci de Azevedo R, Lopes da Silva F, Noronha EF, Ulhoa CJ, Monteiro VN, Cardoza RE, Gutiérrez S, Nascimento Silva R (2015). The Cerato-Platanin protein Epl-1 from *Trichoderma harzianum* is involved in mycoparasitism, plant resistance induction and self cell wall protection. Sci Rep.

[56] Bettini PP, Frascella A, Comparini C, Carresi L, Pepori AL, Pazzagli L, Cappugi G, Scala F, Scala A (2012). Identification and characterization of GEO1, a new class II hydrophobin from *Geosmithia* spp. Can. J. Microbiol..

[57] Brasier CM (1971). Induction of sexual reproduction in single A2 isolates of *Phytophthora* species by *Trichoderma viride*. Nature.

[58] Brasier CM (1975). Stimulation of sex organ formation in *Phytophthora* by antagonistic species of *Trichoderma.* I. The effect in vitro. New Phytol..

[59] Brasier CM (1975). Stimulation of sex organ formation in *Phytophthora* by antagonistic species of *Trichoderma.* II. Ecological implications. New Phytol..

[60] Hänzi M, Cochard B, Chablais R, Crovadore J, Lefort F (2016). First report of *Geosmithia langdonii* and *Geosmithia* spp. isolated from a decaying elm (*Ulmus minor*) in Geneva, Switzerland. Folia Forestalia Polonica.

[61] Martin JA, Witzell J, Blumenstein K, Rozpedowska E, Helander M, Sieber TN, Gil L (2013). Resistance to Dutch elm disease reduces presence of xylem endophytic fungi in elms (*Ulmus* spp.). PLoS One.

[62] Gilbert GS, Parker IM (2010). Rapid evolution in a plant pathogen interaction and the consequences for introduced host species. Evol. Appl..

[63] Lam BS, Strobel GA, Harrison LA, Lam ST (1987). Transposon mutagenesis and tagging of fluorescent *Pseudomonas*: Antimycotic production is necessary for control of Dutch elm disease. Proc. Natl. Acad. Sci. U. S. A..

[64] Myers DF, Strobel GA (1983). *Pseudomonas syringae* as a microbial antagonist against *C. ulmi* in the apoplast of American elms. T Brit Mycol Soc.

[65] Scheffer RJ (1983). Biological control of Dutch elm disease by *Pseudomona*s species. Ann Appl Biol.

[66] Scheffer RJ, Strobel GA, Mukerji KG, Garg KL (1988). Dutch elm disease, a model tree disease for biological control. Biocontrol of plant diseases.

[67] Strobel GA, Lanier GN (1981). Dutch elm disease. Sci. Am..

[68] Webber JF (1981). A natural biological control of Dutch elm disease. Nature.

[69] Webber JF, Gibbs J (1984). Colonization of elm bark by *Phomopsis oblonga*. T Brit Mycol Soc.

[70] Webber JF, Hedger JN (1986). Comparison of interactions between *Ceratocystis ulmi* and elm bark saprobes in vitro and in vivo. T Brit Mycol Soc.

