# Correction to: *Geosmithia-Ophiostoma*: a New Fungus-Fungus Association

**DOI:** 10.1007/s00248-017-1121-9

**Published:** 2017-12-07

**Authors:** Alessia L. Pepori, Priscilla P. Bettini, Cecilia Comparini, Sabrina Sarrocco, Anna Bonini, Arcangela Frascella, Luisa Ghelardini, Aniello Scala, Giovanni Vannacci, Alberto Santini

**Affiliations:** 1 Institute for Sustainable Plant Protection (IPSP-CNR), via Madonna del Piano 10, 50019 Sesto Fiorentino, FI Italy; 2 Department of Biology, University of Florence, via Madonna del Piano 6, 50019 Sesto Fiorentino, FI Italy; 3 Department of Agri-Food Production and Environmental Science (DiSPAA), University of Florence, Piazzale delle Cascine 28, 50144 Florence, Italy; 4 Department of Agriculture, Food and Environment (DAFE), University of Pisa, via del Borghetto 80, 56124 Pisa, Italy

**Correction to: Microb Ecol (2017)**

10.1007/s00248-017-1062-3

The article *Geosmithia-Ophiostoma*: a New Fungus-Fungus Association, written by Alessia L. Pepori, Priscilla P. Bettini, Cecilia Comparini, Sabrina Sarrocco, Anna Bonini, Arcangela Frascella, Luisa Ghelardini, & Aniello Scala, Giovanni Vannacci, Alberto Santini, was originally published electronically on the publisher’s internet portal (currently SpringerLink) on September 5, 2017 without open access.

With the author(s)’ decision to opt for Open Choice the copyright of the article changed on November 2017 to © The Author(s) 2017 and the article is forthwith distributed under the terms of the Creative Commons Attribution 4.0 International License (http://creativecommons.org/licenses/by/4.0/), which permits use, duplication, adaptation, distribution and reproduction in any medium or format, as long as you give appropriate credit to the original author(s) and the source, provide a link to the Creative Commons license and indicate if changes were made. The original article has been corrected.

