# Supplementary figures and images for: Geosmithia-Ophiostoma: a New Fungus-Fungus Association
Source: Microb Ecol. 2017 Sep 5;75(3):632–46. doi: 10.1007/s00248-017-1062-3 (PMC5856884; doi:10.1007/s00248-017-1062-3)

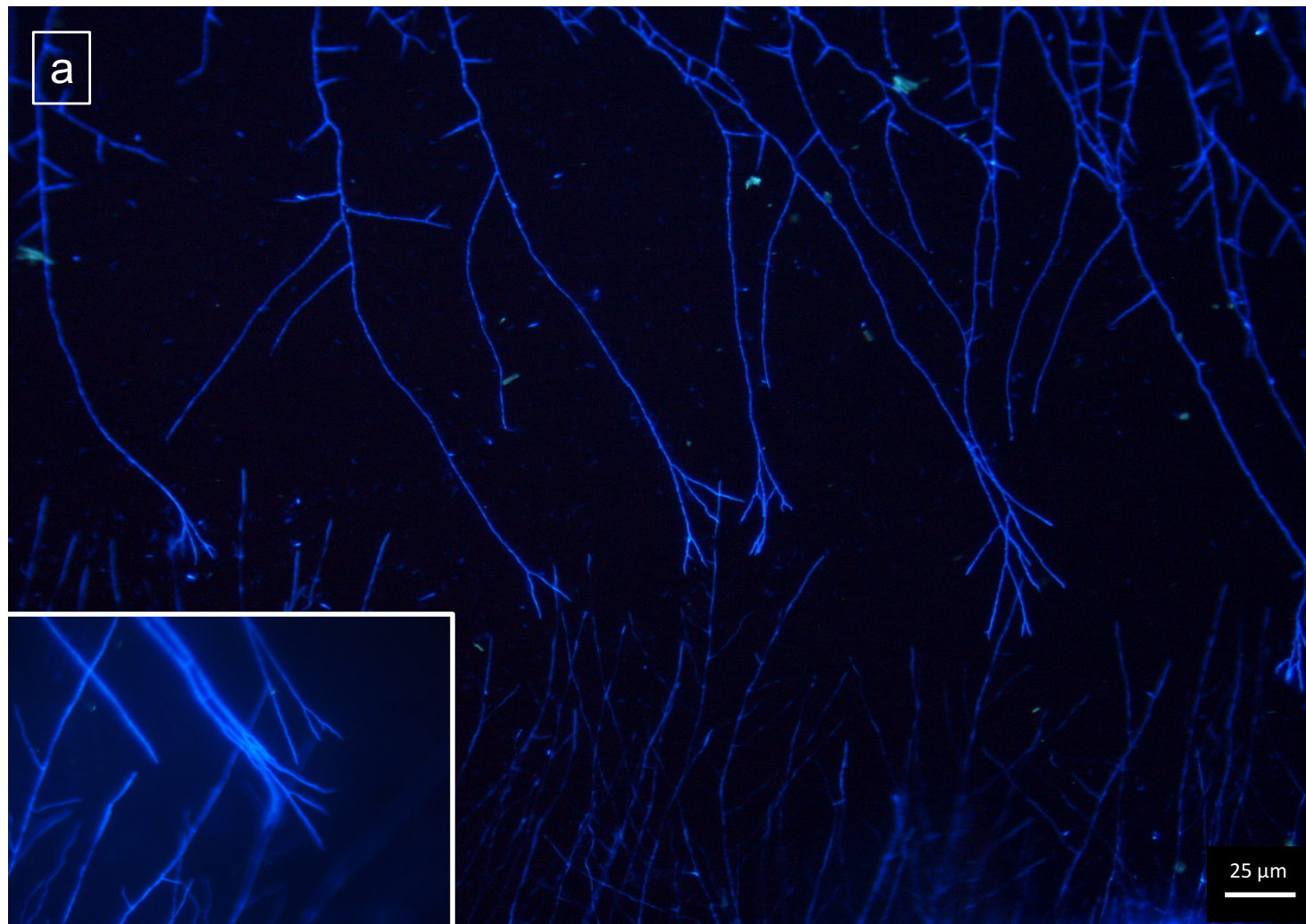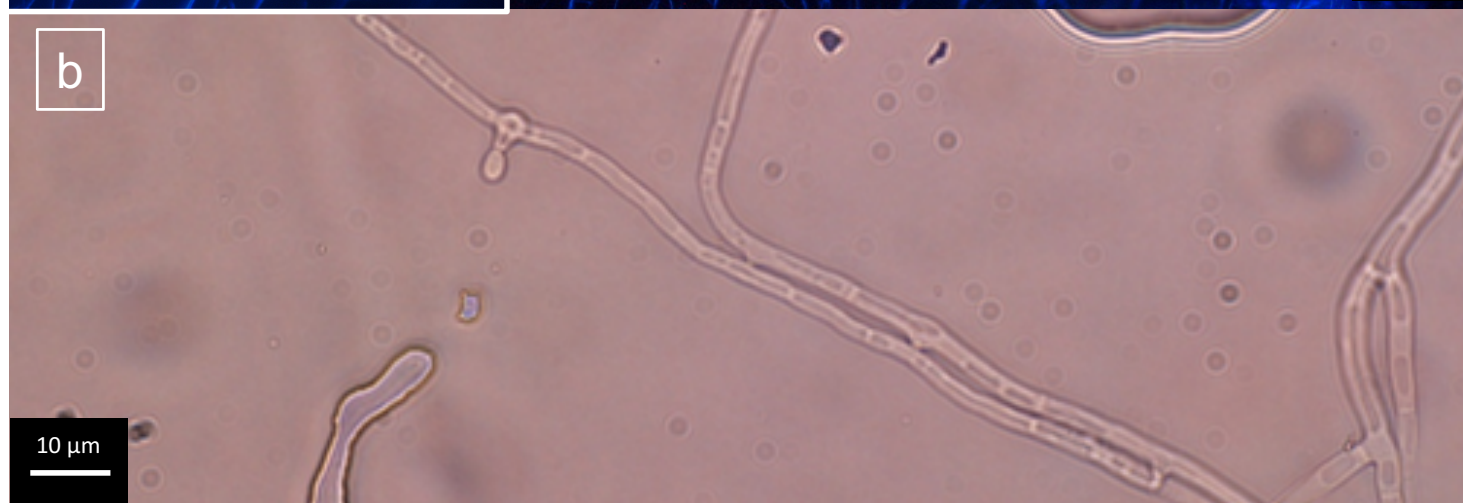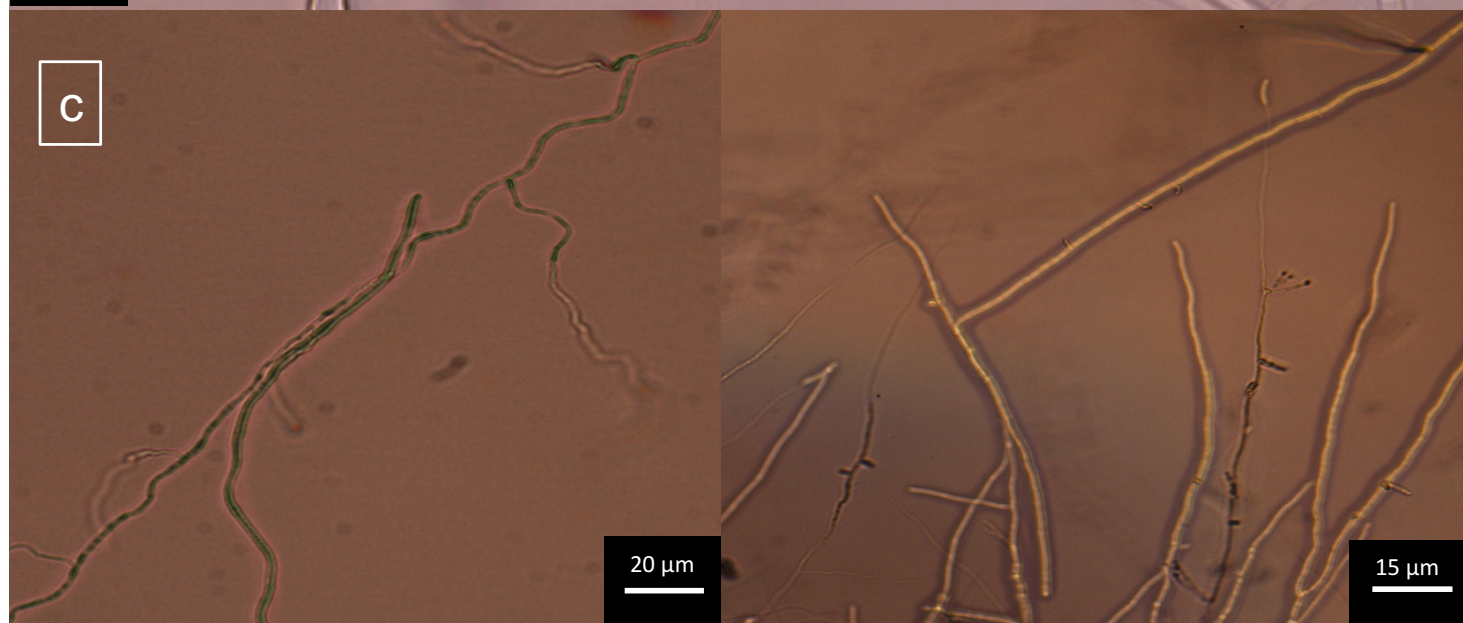

Supplement: Supplementary file 1 — (PDF 1728 kb) [file 248_2017_1062_MOESM1_ESM.pdf]
