# Supplementary material for: Geosmithia-Ophiostoma: a New Fungus-Fungus Association
Source: Microb Ecol. 2017 Sep 5;75(3):632–46. doi: 10.1007/s00248-017-1062-3 (PMC5856884; doi:10.1007/s00248-017-1062-3)
Supplement: Supplementary file 2 — (DOCX 75 kb) [file 248_2017_1062_MOESM2_ESM.docx]

**Title:**

# ***Geosmithia-Ophiostoma*: a New Fungus-Fungus Association**

Alessia L. Pepori^1^, Priscilla P. Bettini^2^, Cecilia Comparini^1,3^, Sabrina Sarrocco^4^, Anna Bonini^3^, Arcangela Frascella^2^, Luisa Ghelardini^1,3^, Aniello Scala^3^, Giovanni Vannacci^4^, Alberto Santini^1^

^1^Institute for Sustainable Plant Protection (IPSP-CNR), via Madonna del Piano 10, 50019 Sesto f.no (FI), Italy

^2^Department of Biology, University of Florence, via Madonna del Piano 6, 50019 Sesto f.no (FI), Italy

**^3^**Department of Agri-Food Production and Environmental Science (DiSPAA), University of Florence, Piazzale delle Cascine 28, 50144 Firenze, Italy

^4^Department of Agriculture, Food and Environment (DAFE), University of Pisa, via del Borghetto 80, 56124 Pisa, Italy

🖂Alberto Santini

[alberto.santini@cnr.it](mailto:alberto.santini@cnr.it)

phone: +39 055 5225586

**Journal:** Microbial ecology

**Supplemental Figure S1** a-c Interacting hyphae of *Ophiostoma* *novo-ulmi* (ONU) and *Geosmithia* sp. 5 ‘IVV7’ in dual culture approaching each other after 24 h incubation (a, Calcofluor white stain 5 mM in water) and forming pseudopod-like structures and short hooks (b, c)
